# Diet and Oral Health: An Investigation Into the Impact of Pakistani Dietary Habits on Oral Disease Prevalence

**DOI:** 10.7759/cureus.71423

**Published:** 2024-10-14

**Authors:** Tayyaba Rafiq, Rummana Aqeel, Mariyah Javed, Lubna Yousaf, Shaher Bano, Aaisha Akbar

**Affiliations:** 1 Department of Oral and Maxillofacial Surgery, College of Dentistry, Lahore Medical and Dental College, Lahore, PAK; 2 Department of Oral Pathology, College of Dentistry, Lahore Medical and Dental College, Lahore, PAK; 3 Department of Oral Pathology, Rahbar College of Dentistry, Lahore, PAK; 4 Department of Community Dentistry, Avicenna Medical and Dental College, Lahore, PAK; 5 Department of Oral Biology, Rahbar College of Dentistry, Lahore, PAK; 6 Department of Orthodontics, Azra Naheed Dental College, Superior University, Lahore, PAK

**Keywords:** dental caries, dietary habits, fruits, oral health, pakistan, periodontal disease, sugar intake, vegetables

## Abstract

Background

Dietary habits play a significant role in the rising prevalence of oral health disorders, such as dental caries and periodontal diseases, particularly in regions like Pakistan with distinct dietary customs.

Objective

The primary objective of this research was to investigate the impact of Asian food on the prevalence of oral diseases, with a focus on identifying specific dietary factors that contribute to oral health issues.

Methodology

A cross-sectional study was carried out in Lahore between February and July 2024. The sample consisted of 384 adults aged 18 and above, selected based on specific inclusion and exclusion criteria. Data were collected through structured questionnaires assessing dietary habits and clinical examinations to diagnose oral diseases. Statistical analyses (SPSS version 26: (IBM Corp., Armonk, NY, USA), including correlation, regression, and Chi-Square, were used to evaluate the relationships between dietary patterns and oral health indicators.

Results

The study revealed significant correlations between dietary habits and oral disease prevalence. Among the 384 patients, 287 (74.74%) patients reported high sugar consumption, with a mean frequency of 3.26 times per week, correlating strongly with oral diseases (r=0.45, p<0.01). This group exhibited 179 patients (46.60%) with dental caries and 153 patients (39.58%) with periodontal conditions. Additionally, 262 patients (68.32%) with high carbohydrate intake, averaging 4.14 times per week, showed a moderate positive correlation with oral disease prevalence (r=0.32, p<0.05). Conversely, 193 patients (50.26%) who consumed fruits and vegetables with a mean frequency of 2.51 times per week demonstrated a significant negative correlation with oral disease prevalence (r=-0.40, p<0.01), indicating a protective effect. High sugar intake was associated with greater severity of oral diseases, with mean severity scores of 3.12 for dental caries and 3.20 for periodontal conditions.

Conclusion

The results show that although consuming more fruits and vegetables has a preventive effect, traditional Pakistani dietary practices, particularly excessive sugar and carbohydrate consumption, are associated with an increased incidence of oral illness.

## Introduction

Dental health plays a crucial role in total health, impacting not only physical health but also social and psychological facets of life [1,2]. Dietary practices have emerged as a critical determinant in the development of oral disorders, including dental caries and periodontal problems, which are becoming more prevalent globally [3]. The varied and deeply ingrained dietary customs in Pakistan have the potential to have a major impact on oral health [4,5]. Siddiqui, et al. reported that nearly 60% of the Pakistani population has dental caries, highlighting the widespread nature of this issue [6]. It is unclear how much of the traditional Pakistani diet, which is heavy in sweets, carbs, and spices, has to do with the frequency of oral diseases in the country's population [7].

A link has been shown between high sugar consumption and a higher incidence of dental caries, underscoring the influence of dietary patterns on oral health [8,9]. There may be an increased risk of dental caries in Pakistan, where sweet foods and drinks are often consumed during meals and special occasions [10,11]. Furthermore, while eating a lot of spiced food is good for you in many ways, it may also harm your teeth via abrasive effects on tooth enamel and changes in pH [11-13].

A thorough investigation of food patterns and their particular impacts on oral health indicators is necessary to comprehend the connection between oral health and dietary habits. The purpose of this study is to provide a thorough examination of the relationship between the incidence of oral illnesses and traditional Pakistani eating patterns. Through an analysis of dietary consumption patterns and the frequency of consuming cariogenic and acidic foods, this study aims to provide new insights into the association between nutrition and the incidence of oral diseases in Pakistan.

Research objective

The primary objective of this research was to investigate the impact of Pakistani dietary habits on the prevalence of oral diseases, with a focus on identifying specific dietary factors that contribute to oral health issues.

## Materials and methods

Study design and setting

This cross-sectional study examined the impact of Pakistani eating practices on the prevalence of oral diseases. Data were collected by the researchers at Chaudhary Muhammad Akram Dental Hospital in Lahore, between February 2024 and July 2024, providing a focused environment for exploring oral health concerns within a specific community. The six-month duration allowed sufficient time for comprehensive data collection and analysis.

Inclusion and exclusion criteria

Patients who were 18 years of age or older, had lived in Lahore for at least the previous year and provided informed consent were the only ones eligible for inclusion. Patients receiving therapies that would interfere with dietary evaluations, those with systemic illnesses or disorders that substantially impair oral health independent of diet, and those unable or unwilling to provide informed permission were all excluded.

Sample size

The World Health Organization method for cross-sectional research was used to determine the sample size. This calculation considered the expected prevalence of oral illnesses, the required degree of accuracy, and the confidence interval. Based on a 50% prevalence assumption (to guarantee a conservative approximation), a 95% confidence level, and a 5% margin of error, the calculation yielded the necessary sample size of around 384 patients.

Data collection

Clinical exams and structured questionnaires were used to gather data. Using a validated food frequency questionnaire, participants were questioned about their eating habits, including the frequency and types of food consumed. Clinical evaluations included oral exams carried out by qualified and calibrated dental practitioners, ensuring consistency and accuracy in identifying and documenting the presence of oral disorders. For dental caries assessment, the Decayed, Missing, and Filled Teeth (DMFT) index was utilized, recording the number of affected teeth as an indicator of caries experience. Periodontal disease was measured using the Community Periodontal Index (CPI), which evaluates periodontal status based on pocket depth, bleeding on probing, and calculus presence. Calibration was conducted through inter-examiner reliability testing before the study commenced, with all examiners achieving acceptable agreement levels. As part of the data-gathering procedure, medical records were reviewed to obtain relevant background information, including participants’ dental history, previous treatments, systemic health conditions, and any medications that may impact oral health.

Statistical analysis

SPSS Software version 26 (IBM. SPSS Statistics, Armonk, NY) was used for statistical analysis. The food habits and demographic data of the patients were compiled using descriptive statistics. Regression and correlation analysis were used to evaluate the association between oral disease prevalence and dietary practices. Chi-square analysis was used to find relationships between the category variables. For statistical significance, a p-value of less than 0.05 was used.

Ethical approval

The study was started after obtaining approval from the Institutional Review Board (IRB) of Lahore Medical and Dental College and every participant gave written informed consent, and their anonymity and privacy were protected at all times throughout the study.

## Results

The demographic details of the research patients (n=384) are shown in Table 1. There are 188 females (48.96%) and 196 males (51.04%) in the sample. The patient's ages are distributed as follows: 170 (44.27%) are in the 18-30 age group, 135 (35.15%) are in the 31-45 age group, 60 (15.63%) are in the 46-60 age group, and 19 (4.95%) are 61 years of age and above. With a standard deviation of ±9.69 years and a mean age of 31.18 years, the research sample exhibits a significantly broad age range.

**Table 1:** Demographic characteristics of study participants (n=384)

Demographic characteristic		Number of patients (n)	Percentage (%)
Gender	Male	196	51.04
	Female	188	48.96
Age group	18-30 years	170	44.27
	31-45 years	135	35.15
	46-60 years	60	15.63
	61+ years	19	4.95
	Mean±SD	31.18±9.69	-

An overview of the patients eating patterns is given in Table 2, which also includes the number of afflicted persons, prevalence percentages, and mean consumption frequencies with standard deviations. According to the data, 287 patients (74.74%) eat sugary meals on average 3.26 times a week ±1.17 days, mostly sweetened sweets and soft drinks. About 262 patients (68.32%) reported consuming a high frequency of carbohydrates (4.14 times per week ±1.39 days), mostly from bread, rice, and potatoes. A total of 231 patients (60.16%) reported consuming spicy food, with an average of 5.36 times ±1.25 days per week. This includes pickles and hot curries. Last but not least, 193 patients (50.26%) emphasized seasonal fruits and salads while consuming fruits and vegetables, with a mean frequency of 2.51 times per week ±0.97 days.

**Table 2:** Dietary habits of participants and their prevalence (n=384)

Dietary habit	Number of patients (n)	Percentage (%)	Mean±SD	Most common food items
Consumption of sugary foods	287	74.74	3.26±1.17	Sweetened desserts, soft drinks
High carbohydrate intake	262	68.32	4.14±1.39	Breads, rice, potatoes
Spicy food consumption	231	60.16	5.36±1.25	Spicy curries, pickles
Fruit and vegetable intake	193	50.26	2.51±0.97	Seasonal fruits, salads

The frequency of oral illnesses among the 384 research patients is shown in Table 3. There were 179 patients with dental caries, accounting for 46.60% of the total, with a mean severity score of 2.78 out of 5. With a mean severity score of 3.16, periodontal diseases afflicted 153 people or 39.58% of the sample. A mean severity score of 2.93 was reported in 138 patients (35.42%) with gingivitis. Of the group, 74 patients (19.27%) experienced oral ulcers, with an average severity score of 2.39. The p-value 0.34 indicates the probability of observing the frequency and severity of oral diseases among the 384 study participants under the null hypothesis, with a p-value less than 0.05 suggesting a statistically significant difference in the prevalence of conditions.

**Table 3:** Prevalence of oral diseases among participants (n=384) A p-value <0.05 is considered significant.

Oral disease	Number of patients (n)	Prevalence (%)	Mean severity score (1-5)	X^2^	df	P-value
Dental caries	179	46.60	2.78	3.36	3	0.34
Periodontal conditions	153	39.58	3.16			
Gingivitis	138	35.42	2.93			
Oral Ulcers	74	19.27	2.39			

The relationship between dietary practices and the incidence of oral illnesses is examined in Table 4. With an r-value of 0.45 and a statistically significant p-value of less than 0.01 for oral disease prevalence, the intake of sugary foods is strongly positively correlated with it. The prevalence of oral disease and high carbohydrate consumption also positively correlated, as shown by an r-value of 0.32 and a p-value of less than 0.05. The prevalence of oral disease and the intake of spicy food have a somewhat positive link (r=0.25, p=0.08), indicating marginal relevance. On the other hand, an r-value of -0.40 and a p-value of less than 0.01 indicate a substantial negative link between fruit and vegetable consumption and the incidence of oral illness.

**Table 4:** Correlation between dietary habits and oral disease prevalence A p-value <0.05 is considered significant.

Dietary habit	Oral disease prevalence (r-value)	P-value	X^2^	df
Consumption of sugary foods	0.45	<0.01	10.24	1
High carbohydrate intake	0.32	<0.05	6.73	1
Spicy food consumption	0.25	0.08	3.02	1
Fruit and vegetable intake	-0.4	<0.01	9.21	1

The relationship between dietary variables and the severity of oral illnesses in the patients is shown in Table 5. With a statistically significant p-value of 0.02, a high sugar intake is linked to a mean severity score of 3.12 for dental caries and 3.20 for periodontal disorders, suggesting a clear correlation between higher sugar intake and more severe oral illnesses. A mean severity score of 2.88 for dental caries and 3.10 for periodontal diseases, with a p-value of 0.05 indicating a significant correlation, is associated with high carbohydrate consumption. A mean severity score of 2.93 for dental caries and 3.05 for periodontal problems is associated with spicy food consumption, as indicated by a significant p-value of 0.07, suggesting a trend toward significance but not reaching conventional levels. Finally, with a highly significant p-value of 0.01 for dental caries and 3.25 for periodontal diseases, poor fruit and vegetable consumption is linked to the highest mean severity ratings, highlighting the preventive role of fruits and vegetables in oral health.

**Table 5:** Association of dietary factors with severity of oral diseases A p-value <0.05 is considered significant.

Dietary habit	Mean severity score (dental caries)	Mean severity score (periodontal conditions)	P-value	X^2^	df
High sugar intake	3.12	3.2	0.02	5.8	1
High carbohydrate intake	2.88	3.1	0.05	3.84	1
Spicy food intake	2.93	3.05	0.07	3.27	1
Low fruit and vegetable intake	3.15	3.25	0.01	6.63	1

The link between background data and measures of oral health is shown in Table 6. The mean caries index and periodontal index for residents of Lahore who have lived there for 1-3 years are 2.70 and 2.60, respectively, with a significant p-value of 0.03. The caries index and periodontal index mean values are higher for those who have lived there for four to six years (3.10 and 3.00, respectively), while the greatest mean scores are found for those who have lived there for seven or more years (3.40 and 3.20, respectively). In relation to the number of dental examinations performed annually, the patients who get 0-1 examinations had the highest mean caries index (3.20) and periodontal index (3.10), both of which show a tendency toward significance with a p-value of 0.07. The average caries index and periodontal index for participants with two to three check-ups are 2.90 and 2.80, respectively, but the lowest mean scores for caries and periodontal conditions are 2.60 and 2.50, respectively, for those with four or more check-ups.

**Table 6:** Background information and oral health indicators

Background information		Mean caries index	Mean periodontal index	P-value
Duration of residence in Lahore (years)	1-3 years	2.70	2.60	0.03
	4-6 years	3.10	3.00	
	7+ years	3.40	3.20	
Frequency of dental check-ups per year	0-1 check-ups	3.20	3.10	0.07
	2-3 check-ups	2.90	2.80	
	4+ check-ups	2.60	2.50	

## Discussion

The present investigation examined the influence of dietary practices in Pakistan on the incidence of oral disorders. The findings indicated noteworthy associations between certain dietary patterns and the outcomes related to oral health. Our results demonstrate that a high intake of carbohydrates and sweet meals is highly correlated with a higher incidence of periodontal diseases and dental caries. In particular, 74.74% of participants reported eating sugary meals on average 3.26 times a week, and 68.32% reported eating a lot of carbohydrates on average 4.14 times a week. The 46.60% prevalence of dental caries and 39.58% prevalence of periodontal issues among the participants indicate a strong correlation with the prevalence of oral diseases. These findings support earlier research that found a connection between increased consumption of sugar and carbohydrates and a greater risk of dental caries and periodontal disease [1,2,14].

Association analysis revealed a substantial positive association (r=0.45, p<0.01) between the intake of sugary foods and the incidence of oral illness. These results are consistent with previous research that highlights the function of sugar in the development of dental caries [15]. A moderate association (r=0.32, p<0.05) was also seen with excessive carbohydrate consumption, which is in line with research suggesting carbs have a role in plaque formation and the development of oral illnesses later on [16].

We also looked at the relationship between eating spicy food and oral illnesses and found a little positive connection (r=0.25, p=0.08). Although the association is not as strong, it implies that the abrasive and pH-altering properties of spices could play a small part in the decline of dental health. This result is in line with other research that suggests eating spicy meals may have an influence on dental health, but that effect is often downplayed in favor of sugar and carbs [17,18].

On the other hand, there seemed to be a preventative impact as fruit and vegetable consumption showed a strong negative connection (r=-0.40, p<0.01) with the frequency of oral illness. This is consistent with other studies showing that a greater fruit and vegetable intake is linked to a decreased risk of oral illnesses because of their high fiber and beneficial nutrient content [19,20]. Increased consumption of fruits and vegetables was associated with significantly reduced mean severity ratings of oral disorders, indicating their potential to mitigate oral health problems.

Oral health indices were also impacted by the length of time spent living in Lahore. Mean caries and periodontal index scores were greater among those who had lived there for seven years or more. This might be due to food exposure that has accumulated over time as well as dental hygiene habits. This result aligns with earlier research indicating that extended use of a cariogenic diet may worsen oral health problems [21]. Our results provide strong proof for the correlation between dietary practices and oral health in Pakistan, conforming to extant literature while accentuating particular dietary elements pertinent to this demographic. Interventions aimed at dietary changes should be investigated in future studies to enhance oral health outcomes.

Study limitations

The cross-sectional nature of this study makes it more challenging to establish causative linkages between the frequency of oral diseases and eating patterns. The accuracy of dietary data may be impacted by memory bias introduced by relying only on self-reported food consumption. The results may not be as applicable to other parts of the world with distinct eating habits due to the study's exclusive emphasis on Lahore. Moreover, even after adjusting for a number of confounding variables, residual confounding resulting from additional lifestyle or socioeconomic factors may still affect the findings. Finally, the study's snapshot design could have missed long-term dietary effects on dental health.

Study strengths

This study uniquely investigates the impact of Pakistani dietary habits on oral health, utilizing a comprehensive methodology that includes validated questionnaires and established clinical indices. By focusing on a specific cultural context, it provides valuable insights into the association between high sugar and carbohydrate intake and the prevalence of oral diseases, while also emphasizing the protective role of fruits and vegetables. The use of statistical analyses strengthens the reliability of the findings, highlighting the urgent need for targeted public health interventions tailored to the dietary practices prevalent in Pakistan.

## Conclusions

This study highlights how important nutrition is in determining oral health outcomes by examining how Pakistani eating patterns affect the prevalence of oral illness. The study identified excessive intake of sugar and carbohydrates as important factors contributing to the increasing prevalence of dental caries and periodontal diseases. On the other hand, consuming more fruits and vegetables seems to provide a shield against oral health issues. These results underline how urgently Pakistan needs focused public health initiatives that support dietary changes to lessen oral health problems. Future studies should keep investigating practical methods for incorporating dietary modifications into more comprehensive oral health programs.
